# Prevalence and Determinants of Suicidal Ideation Among Physicians in Saudi Arabia

**DOI:** 10.3390/healthcare13131632

**Published:** 2025-07-07

**Authors:** Ayedh H. Alghamdi, Mohammed A. Aljaffer, Ahmad H. Almadani, Saleh A. Alghamdi, Hasan R. Alshehri, Akeel A. Alyateem, Refan T. Hashim, Fahad D. Alosaimi

**Affiliations:** 1 Department of Psychiatry, College of Medicine, King Saud University, Riyadh 11451, Saudi Arabia; maljaffer@ksu.edu.sa (M.A.A.); ahalmadani@ksu.edu.sa (A.H.A.); faalosaimi@ksu.edu.sa (F.D.A.); 2 Department of Psychiatry, King Saud University Medical City, King Saud University, Riyadh 11362, Saudi Arabia; refanhashim@gmail.com; 3 SABIC Psychological Health Research and Applications Chair, College of Medicine, King Saud University, Riyadh 12372, Saudi Arabia; 4 Department of Psychiatry, College of Medicine, Imam Mohammad Ibn Saud Islamic University, Riyadh 11564, Saudi Arabia; saalghamedi@imamu.edu.sa; 5 Eradh and Mental Health Complex, Riyadh 12571, Saudi Arabia; hal-shehri@moh.gov.sa (H.R.A.); dr_akeel@hotmail.com (A.A.A.)

**Keywords:** physicians, predictive factors, Saudi Arabia, suicidal ideation, suicidality

## Abstract

**Background:** The mental health of physicians has become a pressing global concern. High rates of depression, anxiety, and burnout are reported in the literature, with each condition linked to reduced job satisfaction, increased medical errors, and ultimately suicidal ideation (SI). Although research on physicians’ mental health is emerging in Saudi Arabia, data on suicidality remain scarce. **Objective:** This study aims to determine the prevalence of SI and its determinants among physicians in Saudi Arabia. **Methods:** A cross-sectional study was conducted with 423 physicians across all medical specialties of all ranks, who were recruited using a convenience sampling technique. The study tool comprised three main sections. The first section included questions regarding sociodemographic factors, lifestyle, and work-related factors. The second section included items on suicidality and the Patient Health Questionnaire-9 to screen for depressive symptoms. The third section included the Brief Resilient Coping Scale (BRCS) to measure the coping mechanisms of the participants. **Results:** SI was disclosed by 9.7% of the respondents, with 0.5% reporting previous suicide attempts. Suicidal ideation was independently associated with low income (OR = 3.94, 95% CI 1.32–11.76, *p* = 0.014) and higher depression scores (OR = 1.09 per point, 95% CI 1.02–1.16, *p* = 0.008). Moreover, knowing a colleague with suicidal behavior (i.e., knowing a colleague who had contemplated suicide or had attempted suicide/died by suicide) was significantly associated with SI among our participants (*p* < 0.001 and *p* < 0.006, respectively). Higher scores on the BRCS, specifically with respect to growing from adversity and actively replacing losses, were linked to lower odds of SI (*p* < 0.001 and *p* < 0.045, respectively). **Conclusions:** Physicians in Saudi Arabia experience an alarming level of SI that is associated with low income and depression. The results of this study underscore the importance of additional research to evaluate the effectiveness of intervention programs designed to enhance mental health support for physicians, encourage adaptive coping mechanisms, foster peer support networks, and combat stigma associated with mental illnesses.

## 1. Introduction

The mental health of physicians has become a growing global concern, with several studies reporting high rates of depression, anxiety, and burnout [1,2,3,4]. Such conditions can have significant consequences, including reduced job satisfaction [5] and an increased likelihood of medical errors [6], culminating in suicidal ideation (SI) and actual suicide [7].

Depression and burnout are among the major challenges faced by physicians. A meta-analysis of 54 studies found a pooled estimate of depression at 28.8% among more than 17,500 resident physicians [8]. Furthermore, the literature shows that rates of psychiatric conditions, especially depression and suicide, are considerably higher in doctors than in the general population [9]. In a cohort of 1354 U.S. physicians, burnout was linked to an 85% rise in the odds of SI, but this association ceased after adjustment for depressive symptoms, suggesting that depression mediates the relationship [10]. Mental health conditions in physicians—such as depression, burnout, and SI—can occur concurrently, exacerbating one another. For instance, a systematic review found a significant association between burnout, depression, anxiety, and SI among physicians [2].

Although numerous studies on physicians have focused on depression and burnout [1,2,3,4], research focusing on suicidal ideation is less common. Suicide is defined as death caused by self-directed injurious behavior with an intent to die as a result of the behavior [11]. A suicide attempt is defined as a nonfatal, self-directed, potentially injurious behavior with an intent to die as a result of the behavior, even if the behavior does not result in injury [11]. Some studies define SI as thinking about, considering, or planning suicide [11], noting that SI could vary in severity from passive ideation to active ideation [12]. It is imperative to state that there has yet to be a set of agreed-upon definitions for these terms. The empirical study of suicidality rests on several complementary frameworks. One approach attributes suicidal behavior to overwhelming psychological pain that eclipses coping resources [12]. Beck’s Hopelessness Theory highlights hopeless expectations about the future as playing a key role in suicidal intent [13]. Another theory is Joiner’s Interpersonal Model, which explains suicidality through the combined effects of thwarted belongingness and the belief of being a burden [14]. These theories explain why mood disorders and occupational exhaustion, along with cultivated hopelessness and social disconnection, are considered potential predictors of SI, which is significantly associated with suicide. For instance, the results of a systematic review indicate that individuals with SI are at approximately three times higher risk of suicide than those without SI [15].

Another systematic review across countries found that physicians are at risk of suicide, with a prevalence of 17% for SI and a 0.5% prevalence of suicidal attempts [16]. These findings highlight the need to assess mental health challenges among physicians, with emphasis on suicidality, in order to develop strategies that ensure their safety and mental well-being.

Furthermore, several factors play a role in suicide. Data in the literature reveal that suicide risk is not evenly distributed among physicians, as certain characteristics seem to be potential predictors of suicidality. Certain sociodemographic factors were found to be associated with a greater suicidal risk; for example, female physicians have a higher risk of suicide than their male counterparts [16]. A study conducted in Egypt found that younger and single physicians and those with psychiatric or chronic medical disorders were at a higher risk for suicidality [17]. The level of income was also found to be a contributor to suicidality, with individuals with lower incomes at higher risk [18]. Physicians in certain specialties exhibit higher suicidal vulnerability, such as anesthesiologists, psychiatrists, general practitioners, and general surgeons [16].

In Saudi Arabia, studies have highlighted conditions such as burnout, depression, stress, and anxiety; however, data on suicidality in the country are limited [5,19,20]. A study conducted on the stress and coping mechanisms of consultant physicians in Saudi Arabia found that 5.8% had passive suicidal thoughts and 0.7% had active ideation in the year preceding survey completion [21]. Despite growing global research on physician mental health [22], data on suicidality among physicians in Saudi Arabia remain scarce. The current knowledge gap merits attention within the Saudi sociocultural context. High religious commitment often provides resilience; at the same time, the religious prohibition of suicide may discourage formal help-seeking behavior [23]. Furthermore, the stigma attached to mental illnesses in Saudi Arabia could suppress disclosure and delay care [24]. Nonetheless, policy responses are emerging, as the Saudi National Mental Health Survey and recent mental health statutes signal growing awareness and an evolving governance framework [25,26,27]. This study aims to investigate the prevalence and predictors of SI among physicians in Saudi Arabia, providing insights to guide mental health interventions, support programs, and policies.

## 2. Materials and Methods

### 2.1. Study Design and Participants

This cross-sectional study targeted physicians practicing in Saudi Arabia. The target population comprised physicians listed with the Saudi Commission for Health Specialties (SCFHS)—Saudi Arabia’s official body for licensing and regulating healthcare professionals and training programs. Physicians in the following ranks were included: consultants, registrars and senior registrars, and service residents. The only exclusion criterion was related to communication barriers, such as physicians whom we could not reach.

The minimum sample size was calculated to be 384, assuming a 50% prevalence for maximum variability, a 95% confidence level, and a 5% margin of error. This figure was then increased by 10% to allow for non-responses, yielding a final sample of 423 physicians.

This study used a convenience sampling technique to recruit participants via work-related network groups on social media, specifically through WhatsApp groups. Data were collected using an online survey distributed electronically. Participants were invited to complete the survey via electronic messages containing a direct access link on three occasions.

The first page of the study tool presented the informed consent form, allowing respondents to proceed to the survey or withdraw. Participation was voluntary and anonymous, and no personal identifiers were collected. All participants provided informed consent before accessing the survey, with anonymity and confidentiality strictly ensured throughout. The study protocol received ethical approval from the Institutional Review Board of the Eradah Complex for Mental Health, Ministry of Health in Riyadh, Saudi Arabia (Registration#: H-01-R-063-14).

### 2.2. Instrument

The online survey was formulated in the English language, and it was based on a literature review of physicians’ suicidality and related factors. The final survey was approved via consensus after conducting a pilot study with 20 physicians from various specialties and verifying the survey’s clarity, length, and usability. The participants in the pilot were not included in the final study’s data analysis.

The survey comprised three main sections. The first section was a questionnaire developed by the research team, including questions regarding sociodemographic factors, history of psychiatric and medical illnesses, and lifestyle and work-related factors.

The second section of the survey included an assessment of suicidality and depression. This section mainly consisted of a set of binary questions (Yes/No) on suicidality, including multiple questions on SI and suicide attempts. The primary outcome of the study (i.e., SI) was assessed using the following question: “During the past 12 months, have you had thoughts of taking your own life?” The same question was used to assess SI in other international studies [10,28].

The English version of the Patient Health Questionnaire-9 (PHQ-9) was also incorporated into this section to screen for depressive symptoms. PHQ-9 comprises nine items (scored 0 = “not at all” to 3 = “nearly every day”), yielding a total score of 0–27. Severity is categorized as minimal [1,2,3,4], mild [5,6,7,8,9], moderate [10,11,12,13,14], moderately severe [15,16,17,18,19], or severe [20,21,22,23,24,25,26,27]. According to the literature, this tool has strong reliability and validity [29].

The third section of the survey used the English version of the Brief Resilient Coping Scale (BRCS) to measure the coping mechanisms of participants. This four-item scale, rated on a 5-point Likert scale (1 = “does not apply at all” to 5 = “applies fully”), measures creative problem-solving, emotional control, growth from adversity, and replacement of losses. Scores range from 4 to 20, with higher values indicating greater resilience. The total score is categorized into three categories: 4–13 indicates low resilient coping, 14–16 indicates moderate resilient coping, and 17–20 indicates high resilient coping. The BRCS had previously been validated with a sample of Saudi health professionals [30].

For our sample, the post hoc reliability verifications yielded Cronbach’s alpha values of 0.88 for PHQ-9 and 0.87 for the BRCS, confirming good internal consistency for both scales [29,30]. PHQ-9 and the BRCS are considered measures in the public domain and can therefore be used in their standardized forms without the need for permission.

### 2.3. Statistical Analysis

Statistical analyses were performed using R software version 4.4.0 (R Foundation for Statistical Computing, Vienna, Austria). The research team summarized categorical variables using frequencies and percentages. Associations between suicidality and other categorical factors were tested with chi-square or Fisher’s exact tests (when expected counts were low). To identify independent predictors of SI, we fitted multivariate logistic regression models, reporting odds ratios (ORs) with 95% confidence intervals (CIs). A two-sided *p*-value < 0.05 was considered statistically significant.

## 3. Results

A total of 423 physicians responded to the online survey. Most participants were male (71.4%), non-Saudi (80.3%), married (87.9%), and had children (92.5%). More than one-third (39%) of the participants were under 40 years old, while many participants had 3–4 children (46.8%), and the majority were Muslims (93.6%). Regarding income, over half earned less than SAR 20,000 (Saudi riyals) (54.6%). Individuals with low income had a statistically significant association with SI (*p* = 0.029), with individuals earning less than SAR 20,000 showing higher SI (12.6%) than those earning SAR 20,000 or more (6.3%). However, no statistically significant differences in suicidal thoughts were found across the following: gender, nationality, marital status, having children, number of children, and religion (Table 1).

The main outcome of interest in this study was SI. We found that approximately 1 in 10 physicians in our study had SI, as 9.7% had thoughts of taking their own life during the past 12 months. Passive death wishes were found in 13.2%, expressed as persistent thoughts of being better off dead within the past 12 months. A small proportion (0.5%) reported a previous suicide attempt.

Table 2 presents the lifestyle and well-being factors among the physicians in this study. Depression severity demonstrated a statistically significant association with SI (*p* = 0.005). A higher percentage of participants categorized as having moderately severe to severe depression had SI (21.7%) compared to those with minimal to moderate depression (8.4%). SI was not significantly associated with the average number of sleeping hours, time spent on moderate-intensity physical activity, or income satisfaction. Furthermore, a history of major medical conditions, a history of psychiatric disorders, and exposure to significant life stressors did not demonstrate a significant association with SI. Lastly, access to mental health services and participation in stress management education were not significantly related to suicidal thoughts.

The PHQ-9 responses revealed significant mental health challenges among the physicians in this study, with a high percentage reporting symptoms of depression, such as “feeling down, depressed, or hopeless” (39.2%) and having “trouble concentrating” (53%). Additionally, 42.8% experienced a lack of interest or pleasure in activities. Remarkably, 9.9% had suicidal thoughts on several days, and 2.8% experienced them nearly every day during the last two weeks. These mental health issues were found to affect work, self-care, and relationships, with 46.8% reporting some difficulty at work, 42.6% in self-care, and 41.4% in maintaining relationships.

In a bivariate analysis, various work-related factors were assessed in relation to SI among physicians (Table 3). A statistically significant association was found between perception of work stress and SI (*p* = 0.024). The results showed that SI was present in 12% of those who agreed or strongly agreed that their work was stressful, 1.6% of those who were unsure, and 6% of those who disagreed with the statement that their work was stressful. Similarly, knowing a colleague who attempted or died by suicide was significantly associated with SI, and even knowing a colleague who was seriously contemplating suicide was significantly associated with SI.

There were no significant differences in the prevalence of suicidal thoughts across the regions of the Kingdom of Saudi Arabia (*p* = 0.706) or job titles (*p* = 0.939). Moreover, no significant association was found between SI and the type of working facility—public, private, or both (*p* = 0.234). Despite the high numbers of on-call shifts and ward patients reported by the participants, there were no significant associations between these factors and suicidal thoughts (*p*-values ranging from 0.477 to 0.818).

An analysis of coping mechanisms and their relationship with SI among physicians is shown in Table 4. Positive coping mechanisms—specifically, growing from adversity and actively replacing losses—were linked to lower odds of SI. As the score of BRCS was calculated for all items, the mean resilient coping score was significantly lower among those who had SI than among those without SI (12.7 versus 14.0, *p* = 0.012). Among the 423 participating physicians, 179 (42.3%) were classified as low resilient copers, 175 (41.4%) were classified as moderate resilient copers, and 69 (16.3%) were classified as high resilient copers. Thus, 83.7% fell within the low-to-moderate range, whereas only 16.3% demonstrated high resilient coping.

**Figure 1:**
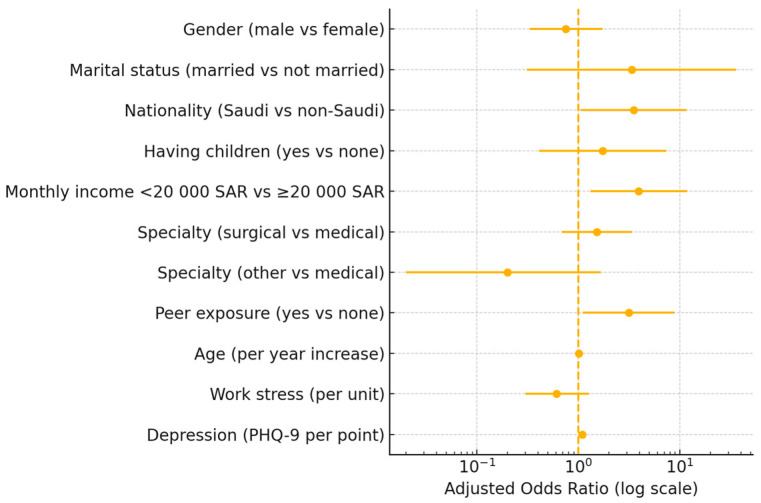
Forest plot of adjusted odds ratios with 95% confidence intervals for predictors of past-year suicidal ideation among physicians.

The multivariate logistic regression model identified several significant predictors of SI among physicians (Table 5). Saudi nationality, earning less than SAR 20,000 per month, knowing that a colleague was contemplating suicide, and higher levels of depression (as measured via PHQ-9) were associated with an increased likelihood of suicidal thoughts. Specifically, Saudis were 3.50 times (95% C.I = 1.05–11.73) more likely to have SI than non-Saudis; those earning less than SAR 20,000 were almost four times more likely to have SI than those earning more, with an odds ratio of 3.94 (95% C.I = 1.32–11.76); and those aware of a colleague contemplating suicide were 3.13 times (95% C.I = 1.10–8.88) more likely to have SI. Additionally, each increase in depression severity raised the odds of suicidal thoughts by 9% (95% C.I. = 2% to 16%). However, factors such as gender, marital status, having children, medical specialty, age, and work-related stress did not show statistically significant associations with SI in the model (Figure 1).

The multivariable logistic equation produced a minus two log likelihood of 193.9, showing an improvement over the intercept-only model. The pseudo-R-squared values were modest, as the Cox and Snell coefficient was 0.070 and the Nagelkerke coefficient reached 0.151. This indicated that the covariates explained roughly 7–15% percent of the variability in past year SI. Calibration appeared to be acceptable because the Hosmer and Lemeshow goodness-of-fit statistic was 10.8, with a non-significant probability of 0.215, suggesting no material difference between the observed and predicted frequencies.

## 4. Discussion

This study is among the early research in Saudi Arabia to explore the prevalence of SI among physicians across all specialties and ranks, while also considering potential associated factors.

Approximately 1 in every 10 physicians in our study reported SI in the past 12 months, while 13.2% had passive death wishes within the same period. The prevalence of SI among the physicians surveyed in our study is approximately five times higher than the Saudi national 12-month average of SI according to the National Mental Health Survey (NMHS), which reported a 1-year prevalence of 1.82% for SI in the community [20]. The higher prevalence of SI among the physicians in our study may be attributed to the fact that physicians and healthcare workers are considered high-risk professions for suicidality [16]. This can be further explained by the fact that the medical field is inherently demanding, with chronic stress, heavy workloads, and disrupted work–life balance [16,19]. Increased suicidality among physicians has serious clinical implications not only for the affected physicians but also for the healthcare system, including in terms of patient care [4]. On the global scale, a systematic review and meta-analysis revealed a pooled 12-month prevalence of 8.6% for SI among physicians, which is similar to the findings in our study [7]. This previous meta-analysis found that doctors had an even higher lifetime prevalence of SI, reaching 17.4% [7]. We did not assess the lifetime prevalence of SI among physicians, which should be explored in future Saudi studies.

In terms of suicidal attempts (SAs), our study found that 0.5% of the participants reported a positive history of attempted suicide, which is lower than the pooled global estimate of 1.8% according to the aforementioned meta-analysis [7]. This difference may be a result of cultural and religious conservativeness in the Kingdom of Saudi Arabia, which contributes to stigma and further impedes the disclosure of suicide attempts [31]. In our sample, only monthly income emerged as a significant predictor of SI. We found that physicians earning less than SAR 20,000 had twice the prevalence of SI than higher earners. This aligns with insights gained from a systematic review of 37 studies, which linked poor economic status, reduced wealth, and unemployment to increased suicidality [18].

The literature indicates that other sociodemographic factors affect suicidality, such as age, gender, and marital status. Our study did not find an association between SI and these factors. In contrast, a study conducted in Egypt found that young and single doctors were at higher risk for suicidal behaviors [17]. Moreover, a cross-sectional study conducted in the United Kingdom (UK) also indicated a high prevalence of SI among junior doctors, affecting about half of the participants [32]. A Norwegian study identified living alone without a partner as a risk factor for both SI and suicide attempts [33]. These diverse findings likely reflect local and methodological differences, such as the narrow age range of the physician samples and the influence of cultural and family support systems. In Gulf societies, extended family networks may provide protective effects for unmarried doctors [34], masking any effect of marital status.

Although we did not find a significant gender difference regarding SI, gender differences in suicide risk have been observed among physicians in numerous studies. A meta-analysis using international data from 1960 to 2020 found a higher age-standardized risk of suicide for female physicians than for women in general [35]. Data from a study in the United States showed that female physicians died by suicide at higher rates than their non-physician counterparts, while the same was not true for their male counterparts [36]. We attribute this difference to equal training rights for women in Saudi healthcare and a supportive work environment, which have been linked to lower burnout rates among female trainees [5]. Future research should gather detailed data on family support, debt burden, and perceived financial strain and should include direct measures of social connectedness. Our results highlight the importance of mental health screening and financial assistance programs targeted at physicians with low income.

Depression remained a strong predictor of SI in our sample, as a 21.7% prevalence rate of SI was identified among physicians with moderately severe to severe depressive symptoms reported versus 8.4% among those with minimal to moderate symptoms. This aligns with previous research showing a robust link between depression and suicidality among clinicians. A 2023 mixed-methods review, including 61 quantitative and qualitative studies, found that most studies reported a significant positive association between depressive symptoms and SI in physicians [2]. PHQ-9, our selected instrument, was one of the most commonly used tools in those studies, reinforcing consistency in comparing measurement tool and results. Likewise, an Egyptian study reported a statistically significant relationship between depression and SI among physicians [17].

Among work-related factors, in our univariate analysis, we found that perceived job stress was significantly associated with SI. Physicians who agreed that their work was stressful had twice the odds of suicidal thoughts compared to those who did not agree. A Saudi study involving clinical consultants similarly found that 6% experienced frequent passive SI over the previous 12 months, using perceived stress measures rather than dedicated suicidality items [21]. Our study also showed that knowing a colleague who was contemplating or had attempted suicide was a risk factor for SI, underscoring the influence of peer exposure to suicide, as reported by the authors of one study [37]. Similarly, in a study conducted on junior doctors in the UK, no clear relationship was found between SI and working conditions [32].

Other workplace variables, such as job title, medical specialty, facility type, on-call shifts, and patient load, showed no independent association with SI. However, a review of factors related to physician burnout and its consequences reported that burnout usually results from excess work-related stressors [4]. Burnout contributors were divided into categories, including work factors (excessive workloads, long working hours, specialty choice, frequent call duties, etc.), personal characteristics, and organizational factors. In the review, some studies reported that burnout leads to a two-fold increase in SI [4].

Our study found no clear link between physicians’ specialty and SI. Similarly, an Egyptian study reported no difference in suicide risk across all specialties [17]. In contrast, a meta-analysis on suicide in physicians found that physicians in some specialties may be at higher risk, such as anesthesiologists, psychiatrists, general practitioners, and general surgeons [16]. These different findings may be related to the small specialty-specific samples, which lack the statistical power to detect differences.

In our study, we used the BRCS to measure coping strategies that may ameliorate SI. Adaptive coping—specifically, growth after adversity and finding ways to replace losses—was associated with a statistically significant reduction in SI. Other studies support the positive effect of coping strategies and resilience on mental health [38,39]. In a study using the BRCS to assess healthcare workers’ resilient coping strategies during COVID-19, the researchers found that higher levels of coping were associated with good to excellent perceived mental health. On the other hand, lower levels of resilient coping were associated with having psychiatric or mental issues and self-isolation [38]. These findings underscore the protective role of resilient coping.

In our multivariate model, four factors emerged as independent predictors of SI, including Saudi nationality, monthly income below SAR 20,000, knowing a colleague who was contemplating suicide, and having moderate-to-severe depression. These results underscore how different socioeconomic and psychological variables intersect to shape suicide risk among physicians.

The association between income and mental health outcomes is not as well-established in the medical profession as in the general population [18]. Physicians usually occupy a high wage bracket, yet they remain susceptible to monetary pressure. For instance, a study in Egypt identified dissatisfaction with salary and incentives as a leading source of job discontent among physicians [40]. Another study of consultant doctors in Saudi Arabia identified financial struggles and low salaries among the most common stressors [21]. However, the specific salary threshold detected in our data is compatible with the relative income hypothesis, which holds that well-being depends less on absolute earnings and more on perceived position within a social reference frame. For practitioners in a wealthy nation, an income judged to be low may evoke feelings of inadequacy, fears of professional underperformance, and difficulty sustaining an expected lifestyle, all of which can foster psychological distress [41]. Our results indicate that Saudi physicians are roughly three and half times more likely than non-Saudi physicians to have suicidal thoughts, even after adjusting for income; this fits the view that, in a wealthy country, earning less than expected can trigger feelings of inadequacy, concerns about work performance, and pressure to maintain a particular lifestyle, which together increase emotional strain. These pressures intersect with the long hours and heavy workload typical of clinical practice in the region, all of which are somewhat linked to anxiety and depression [42]. Furthermore, when financial strain is added to demanding work, the cumulative burden may heighten the risk of suicidal thoughts. Income, therefore, may function as a proxy for perceived success, stability, and status, making it a potent driver of distress in this professional setting. From a policy standpoint, these findings point to an urgent need for targeted interventions in the Kingdom of Saudi Arabia. Initiatives should raise awareness of mental health issues, expand confidential access to psychological support, and mitigate workplace stressors through staffing, workload, and scheduling reforms.

Our study is among the earliest research in Saudi Arabia designed specifically to measure SI among physicians and its associated factors in the Saudi context. Furthermore, this study’s results could greatly aid in illustrating the significance of the topic and, hence, promote the development of strategies and interventions to mitigate the negative consequences of suicidality, especially in this vulnerable population. More specifically, the results could be of great interest to physicians and mental health workers, healthcare policymakers, and other relevant stakeholders in the country.

Our study has certain limitations. The cross-sectional design precludes causal inference. As such, conducting future Saudi studies about the topic using a more rigorous study design, such as a longitudinal study approach, is recommended. Another limitation is that the use of self-reported data may introduce biases, such as recall bias. Future studies could address this limitation by considering an objective assessment of the topic, for instance, using physician-rated assessment scales or structured or semi-structured interviews. A third limitation of this study is the convenience sampling method used, which may limit the results’ generalizability. As such, future studies could use other sampling techniques, such as randomization-based sampling. Moreover, the recruitment of participants in this study through WhatsApp groups might have attracted more digitally engaged or socially connected participants, which might introduce selection bias. That said, targeting closed social media groups restricted to licensed clinicians allowed us to leverage existing professional networks, giving direct access to a large proportion of the practicing physician community while screening out non-eligible respondents. Another limitation of this study is the use of a single dichotomous item to measure SI, which could not provide a reasonable estimate of the test’s internal consistency and might weaken the observed associations. However, we selected one brief question for the assessment of SI, which is common in the literature [10], to minimize respondent fatigue, as this is a common practice in many epidemiological surveys, such the CDC Youth Risk Behavior Survey [43], and the WHO’s World Mental Health Surveys [44]. Hence, for future Saudi studies assessing this topic, we recommend using validated tools for evaluating SI, such as the Beck Scale for Suicide Ideation [45] and the Suicidal Ideation Attributes Scale [46].

The topic of SI in the Saudi context merits further attention and concentrated efforts to mitigate its negative consequences. A analytic review and critical analysis study addressed the topic of advancement toward better mental health in Saudi Arabia and demonstrated that such enhancement is feasible within the current Saudi infrastructure [47]. For instance, annual credential renewal and occupational health visits could include a brief checklist that captures some of the key predictors of SI, as the results of our study indicated. Such a checklist could include items such as monthly income, recent exposure to peer suicide, and screening items for depression, all of which could help identify clinicians at higher risk of SI and, hence, allow earlier intervention. That said, there are some available resources concerning SI in Saudi Arabia. One example of such resources that is readily available to those in training and practicing physicians is the so-called “DAEM,” which translates into “Support” in English, reflecting its valuable mission. DAEM is a mental well-being support program operated by the SCFHS [48].

## 5. Conclusions

Our study found that approximately 1 in 10 physicians in Saudi Arabia had SI. Those earning lower salaries faced higher odds of suicidal thoughts, suggesting that financial hardships undermine psychological well-being. Knowing a colleague who has contemplated suicide increased the risk further, depicting the prominent effect of peer exposure in medical settings. Depression emerged as a powerful predictor of SI, highlighting the importance of easy access to tailored mental health services. By contrast, doctors who employed adaptive coping and active problem-solving and who found growth in adversity were less prone to suicidal thinking.

The results of this study illustrate how economic pressure, workplace culture, psychological distress, and coping style converge to influence SI among physicians in Saudi Arabia. They underscore the critical need to develop and enhance existing support systems and interventional programs that address suicidality. Additional research to evaluate the effectiveness of such programs is also needed in Saudi Arabia. These measures could significantly enhance the mental well-being of physicians, foster their positive adaptive coping mechanisms, strengthen peer support networks, and combat the stigma associated with mental illnesses in the country.

## Figures and Tables

**Table 1:** Sociodemographic characteristics of the physicians based on suicidal ideation in the last year.

Characteristic		Total	Suicidal Ideation (in the Last Year)		*p*-Value
		*F* (% of Total Participants) (*n* = 423)	Yes *F* (% of Participants) (*n* = 41)	No *F* (% of Participants) (*n* = 382)	
Gender	Male	302 (71.4%)	26 (8.6%)	276 (91.4%)	0.234
	Female	121 (28.6%)	15 (12.4%)	106 (87.6%)	
Nationality	Saudi	83 (19.7%)	11 (13.3%)	72 (86.7%)	0.228
	Non-Saudi	338 (80.3%)	30 (8.9%)	308 (91.1%)	
Marital Status	Married	372 (87.9%)	35 (9.4%)	337 (90.6%)	0.594
	Not married	51 (12.1%)	6 (11.8%)	45 (88.2%)	
Have Children	Yes	359 (92.5%)	33 (9.2%)	326 (90.8%)	0.837
	No	29 (7.5%)	3 (10.3%)	26 (89.7%)	
Number of Children	1–2	141 (39.3%)	16 (11.3%)	125 (88.7%)	0.523
	3–4	168 (46.8%)	13 (7.7%)	155 (92.3%)	
	≥5	50 (13.9%)	4 (8.0%)	46 (92.0%)	
Religion	Muslim	396 (93.6%)	41 (10.4%)	355 (89.6%)	0.094
	Non-Muslim	27 (6.4%)	0 (0.0%)	27 (100.0%)	
Income (Saudi Riyals)	Less than 20,000	231 (54.6%)	29 (12.6%)	202 (87.4%)	0.029
	≥20,000	192 (45.4%)	12 (6.3%)	180 (93.8%)	
Age Group	<40	164 (39.0%)	20 (12.2%)	144 (87.8%)	0.055
	40–49	138 (32.8%)	16 (11.6%)	122 (88.4%)	
	≥50	119 (28.3%)	5 (4.2%)	114 (95.8%)	

**Table 2:** Lifestyle and well-being among Saudi physicians.

Characteristic		Total	Suicidal Ideation (in the Last Year)		*p*-Value
		*F* (% of Total Participants)	Yes *F* (% of Participants)	No *F* (% of Participants)	
Average number of sleeping hours per day in the last year	≤6 h	315 (74.5%)	32 (10.2%)	283 (89.8%)	0.589
	>6 h	108 (25.5%)	9 (8.3%)	99 (91.7%)	
In the last year, how much time do you spend on moderate-intensity aerobic physical activity per week?	<50 min/week	251 (59.3%)	28 (11.2%)	223 (88.8%)	0.219
	≥50 min/week	172 (40.7%)	13 (7.6%)	159 (92.4%)	
Are you satisfied with your current income?	Satisfied or strongly satisfied	203 (48.0%)	17 (8.4%)	186 (91.6%)	0.466
	Not sure	81 (19.1%)	7 (8.6%)	74 (91.4%)	
	Dissatisfied or very dissatisfied	139 (32.9%)	17 (12.2%)	122 (87.8%)	
Depression categories based on PHQ-9 score	Minimal to moderate depression	332 (87.8%)	28 (8.4%)	304 (91.6%)	0.005
	Moderately severe to severe depression	46 (12.2%)	10 (21.7%)	36 (78.3%)	
I have a history of major medical illness, including traumatic brain injury.			3 (9.4%)	29 (90.6%)	0.950
I have a current or past history of psychiatric illness.			4 (18.2%)	18 (81.8%)	0.167
I have received professional psychological help.			2 (16.7%)	10 (83.3%)	0.840
I have faced life stressors in the past year.			31 (11.7%)	233 (88.3%)	0.066
I have experienced childhood abuse, neglect, or parental separation.			5 (12.8%)	34 (87.2%)	0.488
I have access to guns, firearms, or other weapons.			2 (15.4%)	11 (84.6%)	0.481
I have been educated or trained in stress management and dealing with burnout phenomena among physicians.			6 (6.8%)	82 (93.2%)	0.306
I find it easy to access mental health treatment in my area.			14 (8.6%)	149 (91.4%)	0.544

**Table 3:** Distribution of work-related factors among physicians with and without suicidal ideation.

Characteristic		Total	Suicidal Ideation (in the Last Year)		*p*-Value
		*F* (% of Total Participants)	Yes *F* (% of Participants)	No *F* (% of Participants)	
Region of practice	Central	86 (20.3%)	8 (9.3%)	78 (90.7%)	0.706
	Western	76 (18.0%)	10 (13.2%)	66 (86.8%)	
	Eastern	45 (10.6%)	4 (8.9%)	41 (91.1%)	
	Southern	102 (24.1%)	11 (10.8%)	91 (89.2%)	
	Northern	114 (27.0%)	8 (7.0%)	106 (93.0%)	
Professional job title	General practitioner	120 (30.2%)	13 (10.8%)	107 (89.2%)	0.939
	Resident and trainee	22 (5.5%)	2 (9.1%)	20 (90.9%)	
	Specialist or consultant	256 (64.3%)	25 (9.8%)	231 (90.2%)	
Medical specialty area	Medical	271 (63.3%)	26 (9.8%)	240 (90.2%)	0.714
	Surgical	123 (28.7%)	13 (10.6%)	110 (89.4%)	
	Others (public health, dentistry, etc.)	34 (7.9%)	2 (5.9%)	32 (94.1%)	
Healthcare facility type	Public (government)	406 (96.0%)	38 (9.4%)	368 (90.6%)	0.234
	Private	7 (1.7%)	2 (28.6%)	5 (71.4%)	
	Both	10 (2.4%)	1 (10.0%)	9 (90.0%)	
Monthly on-call shifts	None	59 (13.9%)	7 (11.9%)	52 (88.1%)	0.818
	1–4	60 (14.2%)	5 (8.3%)	55 (91.7%)	
	5–8	78 (18.4%)	9 (11.5%)	69 (88.5%)	
	>8	226 (53.4%)	20 (8.8%)	206 (91.2%)	
Daily ward patient load	None	66 (15.6%)	3 (4.5%)	63 (95.5%)	0.477
	1–10	107 (25.3%)	11 (10.3%)	96 (89.7%)	
	11–20	86 (20.3%)	10 (11.6%)	76 (88.4%)	
	>20	164 (38.8%)	17 (10.4%)	147 (89.6%)	
Weekly outpatient clinics	None	51 (12.1%)	5 (9.8%)	46 (90.2%)	0.797
	1–4	66 (15.6%)	6 (9.1%)	60 (90.9%)	
	5–8	43 (10.2%)	6 (14.0%)	37 (86.0%)	
	>8	263 (62.2%)	24 (9.1%)	239 (90.9%)	
Do you agree that your work is stressful?	Agree or strongly agree	309 (73.0%)	37 (12.0%)	272 (88.0%)	0.024
	Not sure	64 (15.1%)	1 (1.6%)	63 (98.4%)	
	Disagree or strongly disagree	50 (11.8%)	3 (6.0%)	47 (94.0%)	
During the last 12 months, have you regularly used any of the following:	None	357 (86.7%)	29 (8.1%)	328 (91.9%)	0.541
	Nicotine	44 (10.7%)	7 (15.9%)	37 (84.1%)	
	Benzodiazepines	1 (0.2%)	0 (0.0%)	1 (100.0%)	
	Analgesics	5 (1.2%)	0 (0.0%)	5 (100.0%)	
	Stimulants	4 (1.0%)	0 (0.0%)	4 (100.0%)	
	Opioids	1 (0.2%)	0 (0.0%)	1 (100.0%)	
Do you know a colleague (physician working in Saudi Arabia) who is seriously contemplating committing suicide in the future?		Yes	11 (31.4%)	24 (68.6%)	<0.001
		No	30 (7.7%)	358 (92.3%)	
Do you know a colleague (physician working in Saudi Arabia) who has attempted or died by suicide?		Yes	8 (22.9%)	27 (77.1%)	0.006
		No	33 (8.5%)	355 (91.5%)	
Do you have plans for your future (career, family, finances, etc.)?		Yes	30 (8.5%)	323 (91.5%)	0.062
		No	11 (15.7%)	59 (84.3%)	

**Table 4:** Physicians’ coping strategies assessed via the Brief Resilient Coping Scale.

Characteristic		Total	Suicidal Ideation (in the Last Year)		*p*-Value
		*F* (% of Total Participants)	Yes *F* (% of Participants)	No *F* (% of Participants)	
I look for creative ways to alter difficult situations.	Does not describe me at all	48 (11.3%)	8 (16.7%)	40 (83.3%)	0.344
	Does not describe me	27 (6.4%)	4 (14.8%)	23 (85.2%)	
	Neutral	161 (38.1%)	12 (7.5%)	149 (92.5%)	
	Describes me	134 (31.7%)	12 (9.0%)	122 (91.0%)	
	Describes me very well	53 (12.5%)	5 (9.4%)	48 (90.6%)	
Regardless of what happens to me, I believe I can control my reaction to it.	Does not describe me at all	25 (5.9%)	3 (12.0%)	22 (88.0%)	0.418
	Does not describe me	27 (6.4%)	4 (14.8%)	23 (85.2%)	
	Neutral	149 (35.2%)	18 (12.1%)	131 (87.9%)	
	Describes me	163 (38.5%)	13 (8.0%)	150 (92.0%)	
	Describes me very well	59 (13.9%)	3 (5.1%)	56 (94.9%)	
I believe that I can grow in positive ways by dealing with difficult situations.	Does not describe me at all	25 (5.9%)	4 (16.0%)	21 (84.0%)	<0.001
	Does not describe me	17 (4.0%)	7 (41.2%)	10 (58.8%)	
	Neutral	135 (31.9%)	16 (11.9%)	119 (88.1%)	
	Describes me	182 (43.0%)	11 (6.0%)	171 (94.0%)	
	Describes me very well	64 (15.1%)	3 (4.7%)	61 (95.3%)	
I actively look for ways to replace the losses I encounter in life.	Does not describe me at all	25 (5.9%)	4 (16.0%)	21 (84.0%)	0.045
	Does not describe me	20 (4.7%)	4 (20.0%)	16 (80.0%)	
	Neutral	146 (34.5%)	19 (13.0%)	127 (87.0%)	
	Describes me	181 (42.8%)	12 (6.6%)	169 (93.4%)	
	Describes me very well	51 (12.1%)	2 (3.9%)	49 (96.1%)	

**Table 5:** Adjusted odds ratios for predictors of past-year suicidal ideation among physicians.

Predictor	Reference Category	OR (95% CI) *	*p*-Value
Gender (male)	Female	0.75 (0.33–1.73)	0.501
Marital status (married)	Not married	3.36 (0.31–35.97)	0.316
Nationality (Saudi)	Non-Saudi	3.50 (1.05–11.73)	0.042
Having children (yes)	No children	1.73 (0.41–7.38)	0.457
Monthly income < SAR 20,000 *	≥SAR 20,000	3.94 (1.32–11.76)	0.014
Specialty (surgical)	Medical	1.52 (0.69–3.38)	0.300
Specialty (other)	Medical	0.20 (0.02–1.66)	0.134
Peer exposure to suicidal ideation (yes)	No exposure	3.13 (1.10–8.88)	0.032
Age (per year increase)	—	1.01 (0.97–1.06)	0.559
Perceived work stress (per unit)	—	0.61 (0.30–1.28)	0.193
Depression score (PHQ-9 per point)	—	1.09 (1.02–1.16)	0.008

* Note: OR = odds ratio; CI = confidence interval; SAR = Saudi riyal.

## Data Availability

Data from this study are available from the corresponding author upon reasonable request.
